# Bodymass index and scoliosis among the population of the United Arab Emirates

**DOI:** 10.1186/1748-7161-7-S1-P9

**Published:** 2012-01-27

**Authors:** K Bagnall, T Al Mazrouee, H Al Hamsi, A Al Kaabi, A D’souza

**Affiliations:** 1 UAE University, Al Ain, United Arab Emirates

## Background

Awareness of Adolescent Idiopathic Scoliosis in the United Arab Emirates is not high with even the incidence being unknown although efforts are currently being made to remedy this. Interestingly, the culture and environment has made the Emirati people a closed group and there are some factors within this group that suggest that the incidence of AIS might be higher than in other parts of the world. In this study, the incidence of Body Mass Index among young Emirati women was measured because low BMI levels have been associated with AIS and casual observation has suggested that many young Emirati women have low BMI levels.

## Methods

Standing heights and weights were measured using standard techniques of 100 randomly-selected, young Emirati women and BMI values calculated. The results were compared with similar published data from the United States.

## Results

The results showed that the distribution of BMI values was similar to those in the U.S. but that average height and weight values were both significantly lower.

## Conclusions

These results suggest that the incidence of AIS in the UAE (when determined) will not be expected to be greater than in other parts of the world due to low levels of BMI. Hoewever, normal standards for height and weight in the UAE might need to be adjusted for this specific population in ways similar to some of the Asian countries.

